# Single-cell analyses of transcriptional heterogeneity in squamous cell carcinoma of urinary bladder

**DOI:** 10.18632/oncotarget.11803

**Published:** 2016-09-01

**Authors:** Xiaolong Zhang, Meng Zhang, Yong Hou, Liqin Xu, Weidong Li, Zhihui Zou, Chunxiao Liu, Abai Xu, Song Wu

**Affiliations:** ^1^ Department of Urological Surgery, The Affiliated Luohu Hospital of Shenzhen University, Shenzhen University, Shenzhen, China; ^2^ Shenzhen Following Precision Medical Institute, Shenzhen Luohu Hospital Group, Shenzhen, China; ^3^ Shenzhen Gene Detection Public Service Platform of Clinical Application, Shenzhen Luohu Hospital Group, Shenzhen, China; ^4^ Department of Urology, The First Affiliated Hospital of Anhui Medical University, Hefei, China; ^5^ BGI-Shenzhen, Shenzhen, China; ^6^ Department of Urology, Zhujiang Hospital of Southern Medical University, Guangzhou, China

**Keywords:** bladder cancer, squamous cell carcinoma, single-cell transcriptome sequencing, gene expression

## Abstract

Cell-to-cell expression heterogeneity within a single tumor is a common phenotype among various cancer types including squamous cell carcinoma. To further study the fundamentals and importance of heterogeneity of cell functions and its potential mechanisms, we performed single-cell RNA-seq (scRNA-seq) on human squamous cell carcinoma of the bladder (SCCB) and its corresponding physiologically normal epithelia. Extensive differentially expressed genes were uncovered by comparing cancer and normal single cells, which were preferentially enriched in cancer-correlated pathways, such as p53 signaling and bladder cancer pathway. Furthermore, the most diversely expressed genes were particularly enriched in MAPK signaling pathway, such as *CACNG4*, *CACNA1E* and *CACNA1H*, which involve in cancer evolution and heterogeneity formation. Co-expression network and hub-gene analyses revealed several remarkable “hub genes” of each regulatory module. Some of them are cancer related, such as *POU2F3*, *NKD1* and *CYP2C8*, while *LINC00189*, *GCC2* and *OR9Q1* genes are rarely reported in human diseases. The genes within an interesting module are highly correlated with others, which could be treated as potential targets for SCCB patients. Our findings have fundamental implications for SCCB biology and therapeutic strategies.

## INTRODUCTION

Squamous cell carcinoma of urinary bladder (SCCB), though accounting less than 10% of primary bladder carcinomas, has showed frequent relapse (>50%) and metastasis compared to urothelial carcinoma (UC) [1, 2], highlighting the complex and yet poorly understood mechanisms of SCCB.

Recently, an increasing number of studies have revealed the presence of tumor heterogeneity, which holds a challenge to cancer diagnosis and therapy. It described as modifiability within tumors, and diverse outcomes and therapeutic responses are correlated to different tumor stages, genetic lesions and expression programs [3–5]. Alternatively, cells generated from one tumor commonly comprise distinct mutations, or display diverse phenotypic, or epigenetic status [6–8]. Previous studies identified that UC mostly exhibit mixed histologies within a single tumor, with squamous components being the most common ones [9–12]. However, the definition of SCCB should be reserved exclusively for those with and only with squamous components [13]. And yet for all that, it remains unknown whether intratumoral heterogeneity takes responsibility for the treatment failure and metastasis of SCCBs [14].

Single-cell RNA-seq (scRNA-seq) analysis has been demonstrated to be an efficient method to reveal gene-expression heterogeneity and uncover characteristics of each subpopulation within a tumor at single cell level [15, 16]. In this study, we performed the single-cell tagged reverse transcription (STRT) on a SCCB case and has demonstrated the complexity of the genetic patterns within the tumor, providing a better comprehending of heterogeneous expression profile in SCCB.

## RESULTS

### Clinicopathology of the case

The subject was initially diagnosed with bladder tumor by computed tomography (CT) and further confirmed as SCCB by cystoscope biopsy (Supplementary Figure S1). No other organ metastasis was identified before operation by systematic examination. No neoadjuvant or nor adjuvant chemotherapy was administered pre-operation. Macroscopically, the tumor was 3.0 cm in diameter and located in posterior wall of urinary bladder. After radical cystectomy, histological examination demonstrated that the specimen infiltrated to the outside of the bladder wall and the adjacent intestinal canal was also infiltrated, with no lymph node metastasis found. The patient died six months after the operation because of intestinal metastasis.

### Data description and quality control (QC)

To investigate the intratumoral heterogeneity systematically, we isolated individual cells by Fluorescence Activated Cell Sorting (FACS) from a piece of fresh radically resected and dissociated human SCCB as well as normal urothelial tissues, and performed scRNA-seq based on the STRT method (75 tumor cells, 18 normal cells and 3 negative control) [17]. The mean sequencing depth of all samples is 0.38M reads/sample (ranged from 0.01M∼2.42M), which is sufficient for STRT method to detect gene expression profile. The average mapping rate of all samples is 64.87% (ranged from 28.4%∼91.9%). We attached these details in Supplementary Table S1. In addition, we filtered the cells with detected genes less than 3000, retaining 67 tumor cells and 7 normal cells, respectively (Supplementary Figure S2). Principal Component Analysis (PCA) indicated no normal cell pollution in tumor cells (Supplementary Figure S3). These cells were chosen for further analysis.

### Analyses of differentially expressed genes of cancer

**Figure 1:**
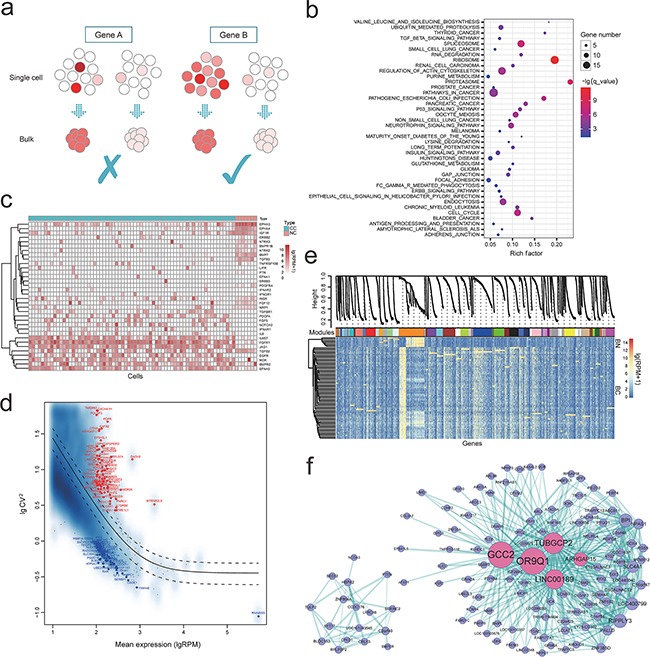
**a.** Bulk approaches were conducted on a “population level” by using the average transcriptomes of millions of cells, frequently fail to uncover the subtle but potentially biologically significant differences between seemingly identical cells, while single-cell transcriptomics will uncover the gene expression at single cell level; **b.** Pathway enrichment of these differentially expressed genes; **c.** Variation in expression of RTKs pathway in tumor and normal single cells population; **d.** Coefficient of variation analyses of these genes highly expressed in cancer cells; according to the variability of these highly expressed genes (RPM > 100), the most 50 variably (red) and most 50 stably (blue) expressed genes were marked, individually; **e.** Gene co-expression modules derived from 74 single cells based on expression level (modules are distinguished by colors); **f.** Hub-gene network of the “darkorange” module in e, and the size of the dots represents hubness. Pink highlights the genes being discussed in the text.

As bulk approaches commonly fail to uncover the subtle but potentially biologically significant differences between seemingly identical cells (Figure 1a), we compared the differences between cancer and normal at single cells level by differentially expressed gene (DEG) analysis using NOISeq (Supplementary Table S2 and Supplementary Figure S4, probability > 0.999) [20]. We noticed many cell cycle correlated genes, such as *CDK1* (↑), *CDC26* (↑), *CCND1* (↑) and *SMAD3* (↓). Cancer is demonstrated as the pathological manifestation of unbounded cell division. And *CCND1* is a crucial regulator of the G1 progression, regarded as a dominant positive regulator of the G1 restriction point [21]. Upregulation of CCND1 was uncovered in various cancers, indicating its potential effects on tumorigenesis process, providing a therapeutic target of this patient. In addition, to further find the pivotal gene sets that function in the tumorigenesis of SCCB, we paid attention to the significant pathways (Figure 1b) that were enriched by DEGs. Compared to the normal cells, we identified that pathways in cancer, p53 signaling, cell cycle and bladder cancer pathways were significantly enriched (Supplementary Figure S5 and S6, Supplementary Table S3). Enormous studies have proved that tumor suppressor gene *TP53* and signaling pathway were imputed to the tumorigenesis and therapy resistance process and, therefore, has been regarded as a vital cellular drug target [22, 23].

### Intra-tumor heterogeneity

We first examined gene expression profile of pathways that have been widely reported in bladder cancer or squamous-cell carcinoma, such as receptor tyrosine kinases (RTKs) (Figure 1c) and epigenetic pathways [24, 25], which are important therapeutic targets for bladder cancer (Supplementary Figure S7) [26, 27], as well as the MAPK, JAK-STAT, Notch, PI3K and VEGF pathways (Supplementary Figure S8-S12). We identified obvious mosaic expression pattern of genes in these pathways, such as *EGFR*, *TGFB2*, *IL6ST*, *BMPR2*, *PDGFA*, *FGFR1*, *FGF12*, *NOTCH2*, *JAG1*, *HSPA9*, etc. These findings indicated that the expression level heterogeneity was existed across individual tumor cells of SCCB, inconsistent with the features of SCCB definition (purity component), which may compromise therapies targeting specific signaling.

Correlations between individual tumor cells from the same tumor showed a broad spread (correlation coefficient *r* ∼ 0.15 to 0.89) (Supplementary Figure S13), consistent with intratumoral heterogeneity. However, no obvious subpopulation was identified within tumor cells. We calculated the coefficient of variation (CV) of each gene to uncover their contribution to intra-tumor heterogeneity (mean RPM > 10, Figure 1d). According to the variability of these highly expressed genes (RPM > 100), we extracted the most 100 variably and most 100 stably expressed gene sets, individually for further analyses. Recent studies showed that cell cycle is a confounding factor of expression heterogeneity [28, 29]. As expected, the variably expressed gene group contains many cell cycle related genes. In addition, six genes were significantly enriched in MAPK signaling pathway (*RPS6KA1*, *RAC2*, *CACNG4*, *CACNA1E*, *CACNA1H* and *MAPKAPK5*, *p* = 1.93×10^−5^, FDR = 3.59×10^−3^), which was identified as a pivotal pathway for human cancer cell survival, dissemination and resistance to drug therapy [30], suggesting the potential role of this pathway on the intratumor heterogeneity formation of SCCB. In contrast, majority of these stably expressed genes were housekeeping genes and enriched in ubiquitin mediated proteolysis, proteasome pathways, results consistent with our expectations.

### Gene co-expression network analysis

To understand the co-expression profile between genes at a system level, we performed Weighted Gene Co-expression Network Analysis (WGCNA) using the expression profile of all single cells [31]. We selected 5530 genes with high variability (mean RPM > 10, SD > 100) for co-expression analysis, and detected 48 different co-expressed modules (Figure 1e). We focused on the top 5 largest modules (“darkorange”, “blue”, “brown”, “yellow” and “green”) for further analysis. Several widely reported cancer related pathways were uncovered within the five modules, such as pathways in cancer, VEGF and MAPK signaling pathways. The largest module (“darkorange”) containing highly differentially expressed genes between cancer and normal single cells, was enriched in both cancer and cell cycle pathways (*p* = 6.9×10^−6^, FDR = 5.95×10^−5^, Supplementary Figure S14, Supplementary Table S4) while the second largest module (“blue” in Figure 1e) were dominated by the neurotrophic signaling pathway (*p* = 7.27×10^−9^, FDR = 1.35×10^−6^), spliceosom (*p* = 1.02×10^−7^, FDR = 9.45×10^−6^) and pathways in cancer (*p* = 4.76, FDR = 3.49×10^−6^, Supplementary Figure S15, Supplementary Table S4). In addition, the pathway enrichment of the other three modules were presented in Supplementary Figure S16-S18 and Supplementary Table S4.

### Hub-gene-network analysis

Hub-gene-network analysis of these modules demonstrated hierarchical organizations of highly connected genes in individual modules, through which key controlling (hub) genes in the modular network can be uncovered. Several significant hub genes of the “darkorange” module were uncovered, such as *GCC2* (↓), *OR9Q1* (↓), *TUBGCP2* (↓), *LINC00189* (↓) and *ARHGAP15* (↓) (Figure 1f). With these genes rarely reported in previous cancer studies, it remains an intriguing question for future research. We recognized *LINC00189*, is one of the major regulators of “darkorange” module network, potentially participating in the tumorigenesis of SCCB. In addition, ArhGAP15 is a potential regulator of Rac1, a member of the Ras superfamily of GTPases involved in signaling pathways controlling cell proliferation and apoptosis [32]. Previous study identified that ArhGAP15 gene knocking out influences the apoptosis induced by ethanol in bovine fibroblast cells [33].

On the other hand, “hub genes” of four other modules contain *POU2F3* (↑), *CENPH* (↑), *PCSK6* (↑) and *HERC2* (↑), *BCAR3* (↑), *DOHH* (↑), *NLK* (↑), *SCN2A* (↑), *CACNA2D3* (↓) and *CTNND2* (↓), which were commonly reported related to cancer (Supplementary Figure S19). Especially, POU2F3 is a keratinocyte-specific POU transcription factor expressed in stratified squamous epithelia [34, 35], and its expression is tied to squamous epithelial stratification [36]. POU2F3 activates genes encoding cytokeratin 10 and SPRR2A, POU2F3, promoting keratinocyte proliferation and enhancing keratinocyte differentiation, and subsequently contribute to epidermal stratification [34, 37, 38]. Besides, down-regulation of POU2F3 was reported correlated to the process of both cervical intraepithelial neoplasia (CIN) and cervical cancer (CC) [39].

## DISCUSSION

In the present work, we conducted scRNA-seq of individual cells generated from one tumor-normal paired SCCB case. We observed obvious differences in expression patterns of genes and pathways between cancer and normal single cells, which may contribute to SCCB processing. We revealed the intratumor heterogeneity within SCCB single cells in expression, and considered that genes encompassed in MAPK signaling pathway involved in cancer evolution and heterogeneity formation. With the combination of co-expression network modules of SCCB single cells and “hub gene” analysis, several “hub genes” were identified, such as *POU2F3* (↑), *NKD1* (↑) and *ARHGAP15* (↓), and the expression networks could collapse when their expression was interfered.

As bulk approaches, which conducted on a “population level” by using the average transcriptomes of millions of cells, frequently fail to uncover the subtle but potentially biologically significant differences between seemingly identical cells. Single-cell transcriptomics will contribute to the reconstitution of temporal transcription networks during developmental processes [40] or when cells are exposed to external stimuli [29], either of which can be masked at the population level. Here we demonstrated the significant DEGs between cancer and normal single cells population, and identified several interesting pathways, including pathways in cancer, cell cycle and P53 signaling pathways. Cancer is usually described as a pathological manifestation of uncontrolled cell division [41]. We noticed several cell cycle related genes such as *CDK1* (↑), *CCND1* (↑) and *SMAD3* (↓), that have been widely investigated in various cancers. Therefore, it has been anticipated for long that our understanding of the basic principles of cell cycle control would lead to effective cancer therapies.

WGCNA is used to uncover the gene co-expression modules and therapeutic targets in SCCB. Several computational methods have been proposed for combining biological pathway information and gene sets into transcriptome data analysis, such as gene set enrichment analysis (GSEA) [42]. WGCNA shares the philosophy of GSEA to concentrate on gene sets as against to individual gene, and gene sets of the modules are tracked back to the RNA-seq data by applying unsupervised clustering. The expression profiles of intramodular hub genes within an interesting module are highly correlated to dozens of targets (in present data, r^2^ > 0.999). Although these targets are statistically equivalent, they may differ biologically. In our data, we revealed a batch of interesting “hub genes” of each co-expression module, including *GCC2* (↓), *OR9Q1* (↓), *LINC00189* (↓), *ARHGAP15* (↓), *POU2F3* (↑) and *NKD1* (↑) [39, 43–45]. Some of them were commonly investigated in cancer, while others were rarely reported, such as *GCC2* and *LINC00189*, which may hold significant role in regulating specific module networks. Of these hub genes, we noticed POU2F3 is reported as a pivotal factor in the complex regulatory network of differentiation, especially tied to squamous epithelial stratification [36]. In addition, *POU2F3* was also reported might be a cancer-related tumor suppressor in both intraepithelial neoplasia and cervical cancer [39], highlighted the importance of *POU2F3* in bladder squamous cell carcinoma. However, further functional validations are necessary for these hub genes, such as RNAi tests, biomarkers availability tests and druggability tests. Comprehending how broadly cancer-related modules interact with specific molecular lesions in an individual cancer patient is critical to identify new molecular targets.

Single-cell transcriptomics offers us unprecedented chance to master the transcriptional stochasticity and cellular heterogeneity, which are important for maintaining cell functions and for promoting disease progression or treatment response, while these details are commonly masked in bulk-cell studies. STRT is a PCR-based multiplexed scRNA-seq method performed on the Illumina platform, regarding as a good method to detect single cell expression profile in a high throughput and low cost way offered by the early barcoding strategy. However, because STRT quantifies transcripts through reads mapping to 5′ ends of mRNA [46], the data could not be used to reveal genetic variations, such as somatic point mutations and structural variations (gene fusion, alternative splicing, etc.). Several other limitations should also be illustrated as follow: 1) the veracity. Single cell RNA-seq comprises sequential steps of target cell isolation, RNA extraction, fragmentation, and reverse transcription. Each step introduces biases and artifacts that may influence the coverage, accuracy, and timeliness of transcript expression and therefore disrupt both the internal characterization and quantification of transcripts. It is therefore important to control the quality of the data prior to including the datasets in a meaningful global study; 2) owing to the limitation of STRT method (basing on the STRT method, we barcoded mRNA of each cell and reversed them into cDNA respectively, and then perform cDNA amplification after pool them together), we cannot validate specific genes identified by the analyses from the amplification products by qPCR. Currently, the STRT method was modified by introducing “unique molecular identifiers (UMI)” and implying on a microfluidic platform (Fluidigm C1 AutoPrep), which substantially decreased the bias of PCR and increased the stability of RNA amplification [47]. Recently, Simone Picelli *et al.* [48] optimized the method of Smart-seq named Smart-seq2 in terms of improved sensitivity, accuracy and full-length coverage across transcripts and decreased cost. Furthermore, Tamar Hashimshony *et al.* [49] made significant improvements that makes CEL-Seq2 uniquely suited to scRNA-Seq analysis in terms of economics, resolution, and ease of use. In addition to external molecule controls, improved single-cell chemistry and physics [50, 51], incorporation of molecular barcoding system [18] have significantly decreased the noise level within each study.

Taken together, we have leveraged single cell RNA-Seq to characterize heterogeneous gene expression profiles within a SCCB case and interrelated their transcriptional, functional, and genetic diversity. In addition, “hub gene” networks are uncovered by WGCNA, a powerful method in providing useful information associated with cell-type specificity. The discovery of these modules should lead to a better comprehension of the molecular features of different cell types along the differentiation and evaluation of SCCB in the future. These findings are to incur fundamental recalibration on tumor biology and therapeutic strategies.

## MATERIALS AND METHODS

### Human tumor specimens

Surgically resected squamous cell carcinoma of urinary bladder (SCCB) specimens were collected at Shenzhen Second People's Hospital with approval by the Institutional Review Board. The fresh tissues (cancer and normal control specimens from one patient) were minced (5-10mm in maximum dimension) during surgery, and kept in cryopreservation medium (10% DMSO+90% DMEM medium with 30% FBS) under -80°C.

### Cell isolation and mRNA sequencing

5 mL of STRT buffer (20 mM Tris-HCl at pH 8.0, 75 mM KCl, 6 mM MgCl2, 0.02% Tween-20) with 400 nM STRT-V3-T30 and 1:50,000 Life Technologies ERCC Spike-In Mix 1 were added into each well of the 96-well plate. The frozen tissues were thawed and digested into single cell suspension using 0.1% collagenase I (1mg/ml, 200U/ml) and 0.05% collagenase IV (0.1mg/ml, 20U/ml) for 2 hours under 37°C. Both single tumor cells and normal cells were sorted into the 96-well plate by Fluorescence-activated cell sorting (FACS) (75 tumor cells and 18 normal cells, 3 negative control). All of the following STRT steps including cDNA amplification and library construction were according to the protocols set by Saiful Islam *et al.* [17] The final cDNA library was sequenced on an Illumina HiSeq2000 using the customized sequencing primer STRT-SEQ-V3. Single-end reads of 50bp were generated along with 8-bp index reads corresponding to the cell-specific barcodes. The sequencing data from this study have been submitted to the NCBI Sequence Read Archive (http://www.ncbi.nlm.nih.gov/sra) under accession no. SRP078083.

### Data processing

Data analysis pipeline was basically followed the previous methods [18]. Generally, raw reads were separated into different FASTQ files by barcode, and trimmed the low-quanlity bases and polluted adapter sequence, and reads that less than 25 bp or contain more than 6 sequential “A” at 3′ site. The “valid STRT reads” were mapped to the human genome (GRCh37, http://www.ncbi.nlm.nih.gov/projects/genome/assembly/grc/) using Tophat 2.0.12 with default parameters expect “--bowtie1”. Unmapped reads were discarded. Then, a homemade Perl script was used to count the reads that align to exons and in the sense orientation toward the expression value for each annotated feature in the NCBI 37.1 assembly. Finally, the reads count of each gene were normalized to Reads Per Million (RPM), which is different from the commonly used RPKM measure that we do not divided reads count by the gene length, because each mRNA gives rise to a single sequenceable fragment near its 5′ end. To exclude poor quality cells, single cells with expressed genes more than 3000 were retained for downstream analysis.

### Differential expression analysis

The DEG analysis was performed between tumor cells and normal cells using NOISeq with recommended parameters (“noiseqbio” function), which can take the RPM matrix as input [19]. Genes with probability > 0.999 were extracted as confident differentially expressed genes. The “MD plot” were generated using the “DE.plot” function of NOISeq with parameters “*q* = 0.999, graphic = “MD”)”.

### Heterogeneity analysis of single cells

Cell-to-cell similarity was quantified as Pearson's Correlation Coefficient, and the variability of gene *A* expression among single cells were estimated as the coefficient of variation (CV), which was generated as the standard deviation of gene *A* expression divided by the mean expression of gene *A* among single cells.

### Co-expression network analysis

To minimize the influence of the stochastic differences during the experimental and sequencing procedures, genes with a mean of RPM less than 10 or a variance of RPM less than 100 were filtered out. The co-expression networks were constructed from the single cells using the weighted co-expression network analysis (WGCNA) R package. Soft power parameter was estimated and used to derive a pairwise distance matrix for selected genes using the topological overlap measure, and the dynamic hybrid cut method with minimum module size of 30 genes was used to detect clusters. The node centrality, defined as the sum of within-cluster connectivity measures, was used to rank genes for “hubness” within each cluster. Hard threshold of edge distances were set separately to limit the numbers of edges and nodes, and the constructed network of each module were denoted using Cystoscape 3.2.1.

## SUPPLEMENTARY MATERIALS FIGURES AND TABLES










