# Supplementary material for: Single-cell analyses of transcriptional heterogeneity in squamous cell carcinoma of urinary bladder
Source: Oncotarget. 2016 Sep 1;7(40):66069–76. doi: 10.18632/oncotarget.11803 (PMC5323215; doi:10.18632/oncotarget.11803)
Supplement: Supplementary file 1 [file oncotarget-07-66069-s001.pdf]

Single-cell analyses of transcriptional heterogeneity in squamous cell carcinoma of urinary bladder

SUPPLEMENTARY FIGURES AND TABLES

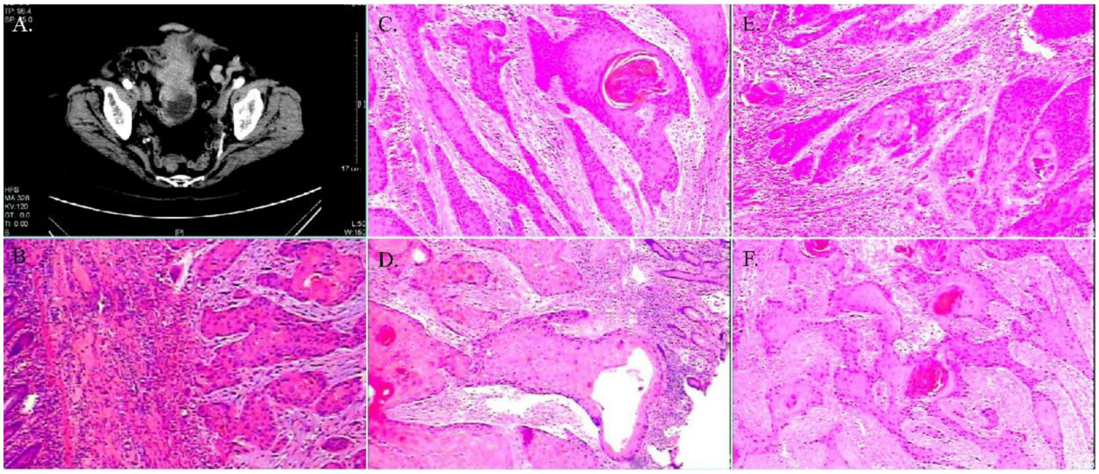

Supplementary Figure S1: Histology of squamous cell carcinoma of urinary bladder case used in this study. The tumor was confirmed located in posterior wall of urinary bladder by computed tomography (CT) **A.** and further verified as squamous cell carcinoma of urinary bladder by cystoscope biopsy **B & C & D & E & F.**

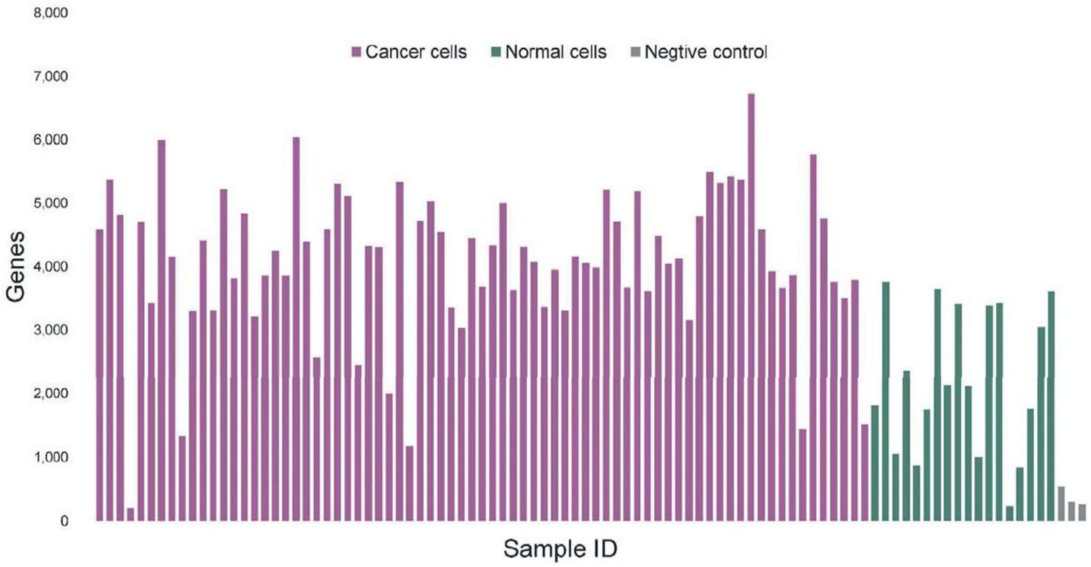

Supplementary Figure S2: The number of detected genes of each cell.

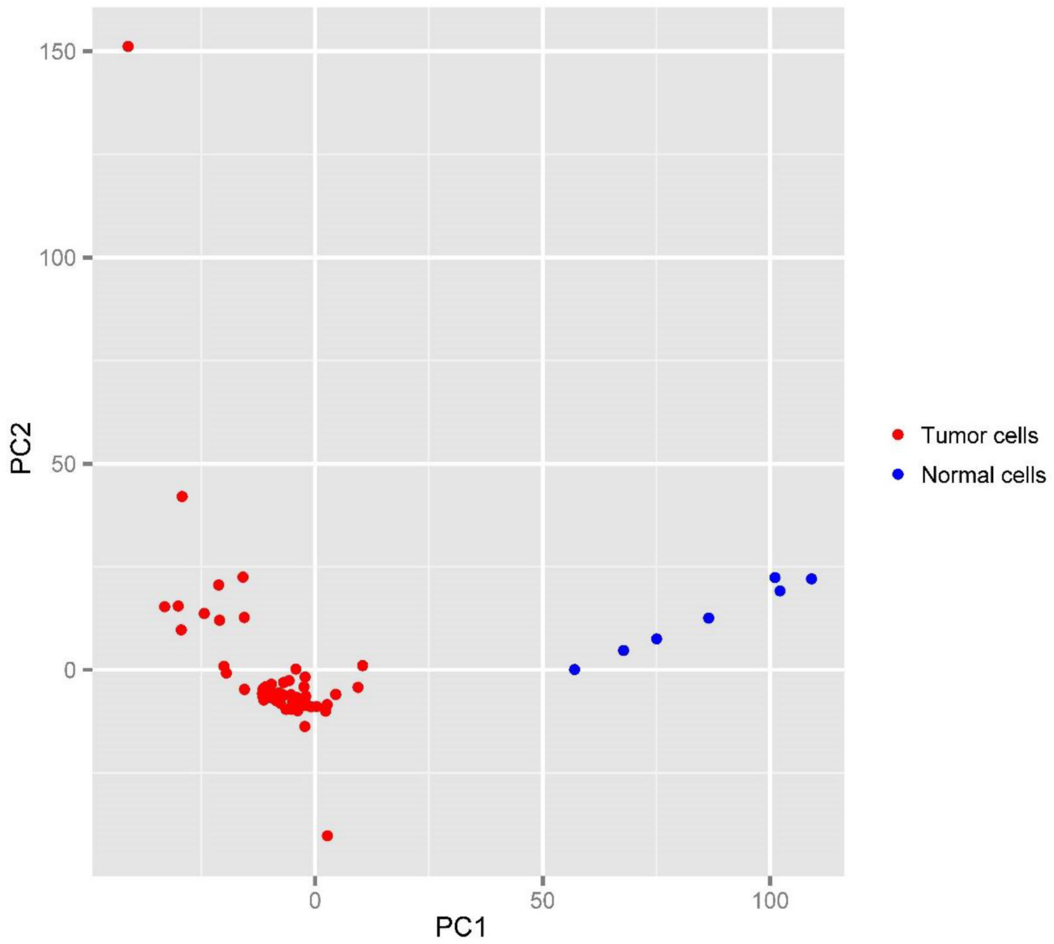

Supplementary Figure S3: Principal components analysis (PCA) of the tumor and normal cells.

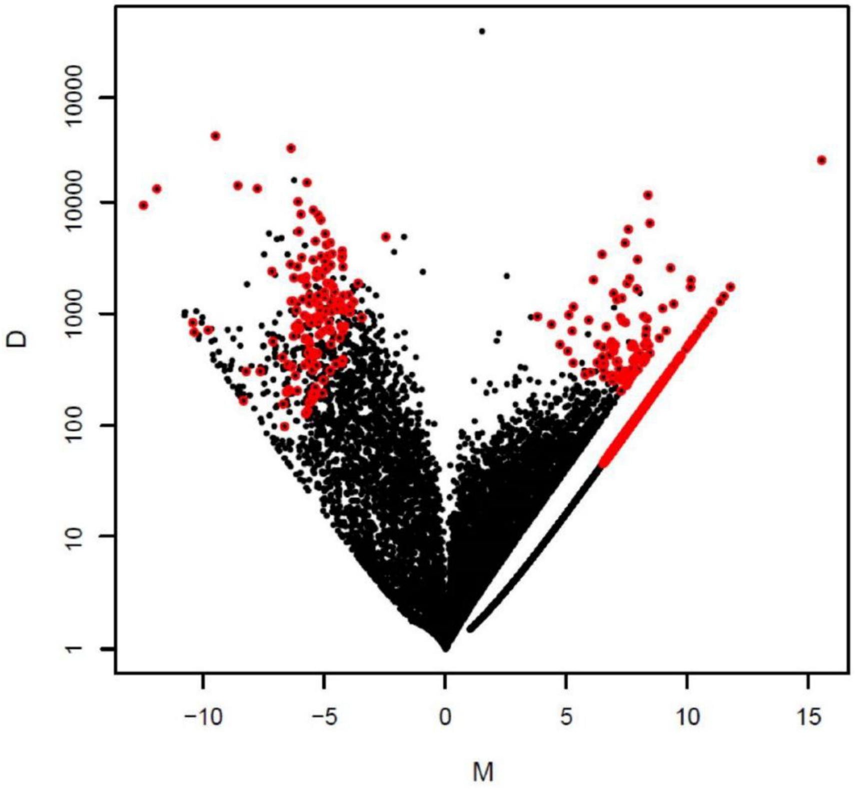

Supplementary Figure S4: MD plot for the differentially expressed genes. "M" indicates the log-fold change and value "D" indicates the difference in expression between cancer single cells and normal single cells. Significant DEGs are marked with red circles.

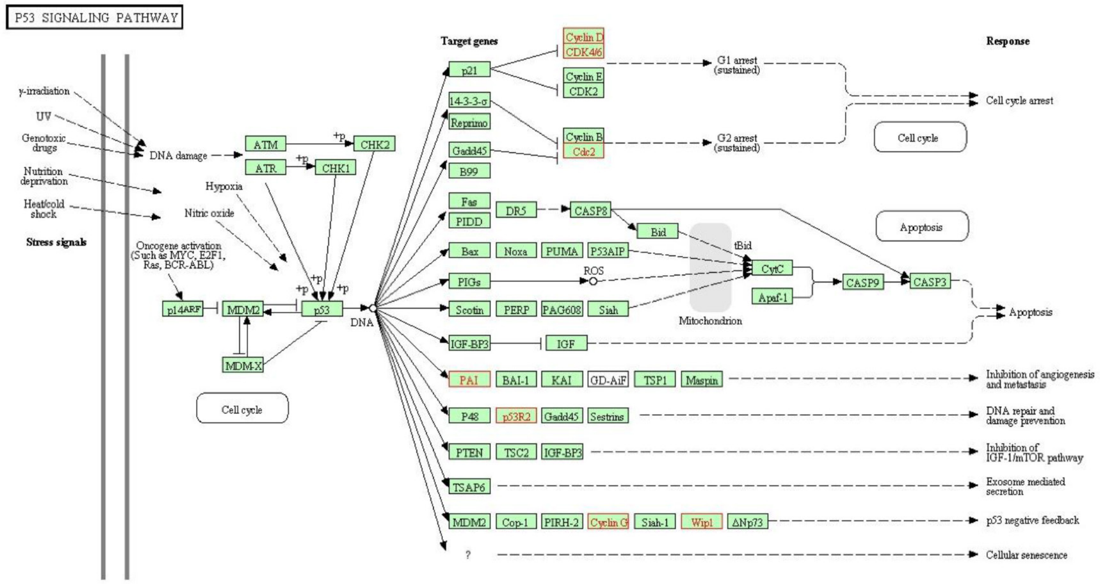

Supplementary Figure S5: KEGG p53 signaling pathway. The differentially expressed genes are denoted by red colors.

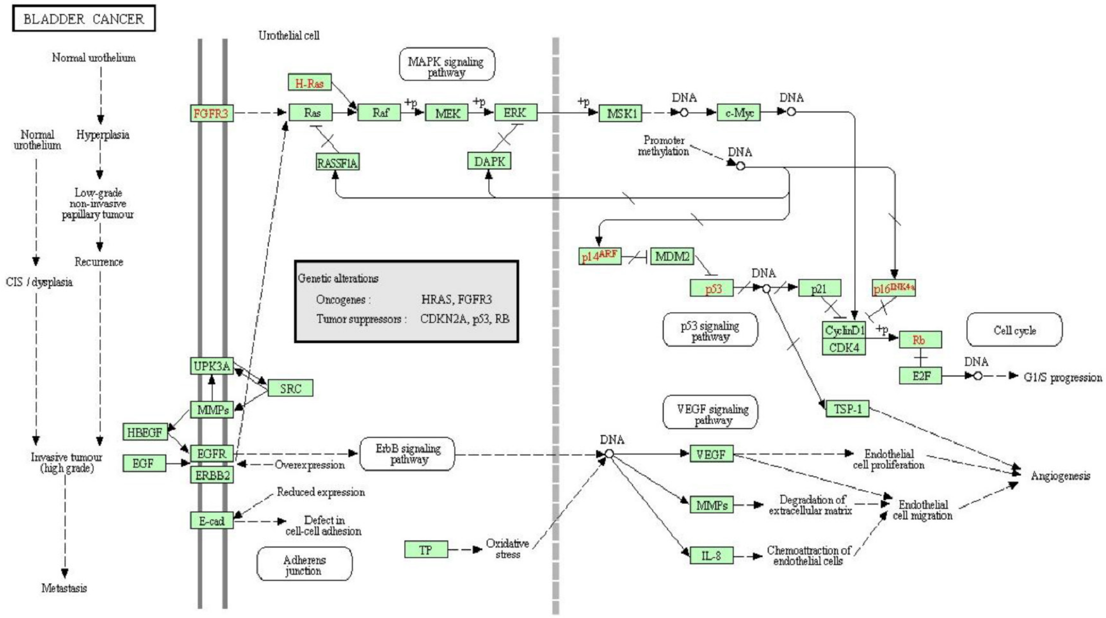

Supplementary Figure S6: KEGG bladder cancer pathway. The differentially expressed genes are denoted by red colors.

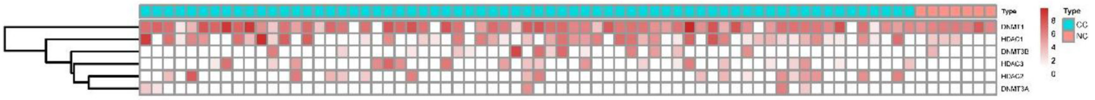

Supplementary Figure S7: Variation in expression of epigenetic pathway in tumor and normal single cells population. Heatmaps for each tumor show expression values for epigenetic pathway (rows, alphabetically ordered with receptors and ligands paired) in each cell (columns).

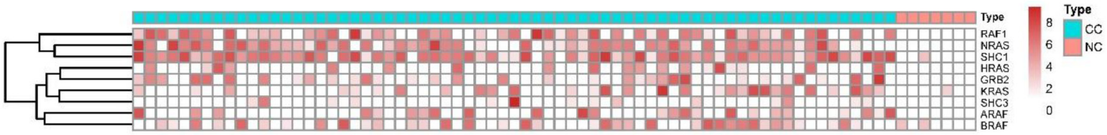

Supplementary Figure S8: Variation in expression of MAPK signaling pathway in tumor and normal single cells population. Heatmaps for each tumor show expression values for MAPK signaling pathway (rows, alphabetically ordered with receptors and ligands paired) in each cell (columns).

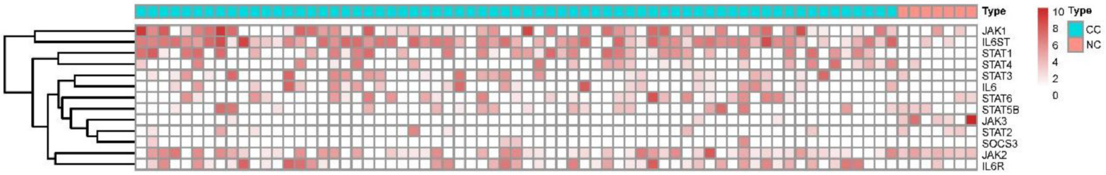

Supplementary Figure S9: Variation in expression of JAK-STAT signaling pathway in tumor and normal single cells population. Heatmaps for each tumor show expression values for MAPK signaling pathway (rows, alphabetically ordered with receptors and ligands paired) in each cell (columns).

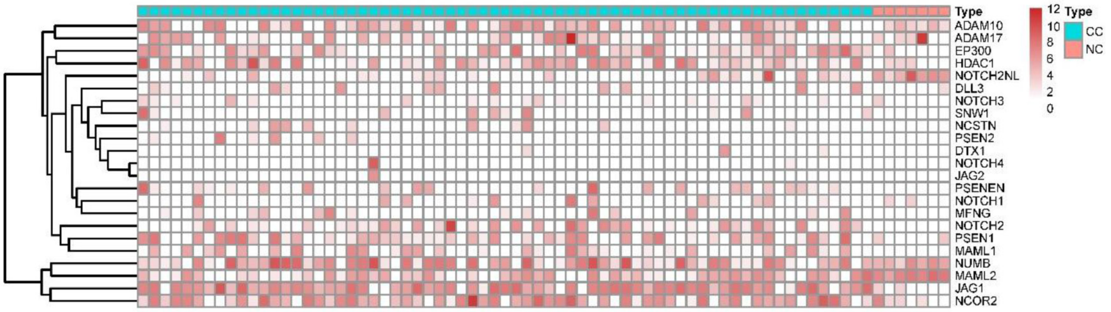

Supplementary Figure S10: Variation in expression of Notch signaling pathway in tumor and normal single cells population. Heatmaps for each tumor show expression values for Notch signaling pathway (rows, alphabetically ordered with receptors and ligands paired) in each cell (columns).

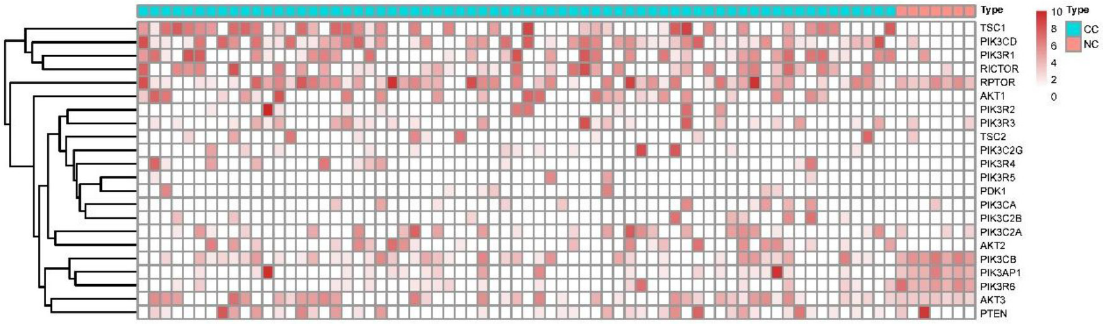

Supplementary Figure S11: Variation in expression of PI3K Signaling pathway in tumor and normal single cells population. Heatmaps for each tumor show expression values for PI3K signaling pathway (rows, alphabetically ordered with receptors and ligands paired) in each cell (columns). All receptors and ligands represented among the 5,948 genes in the filtered dataset are included. Their expression varies considerably in each tumor. Bar plots represent the sum of the signal in rows (genes) and columns (cells).

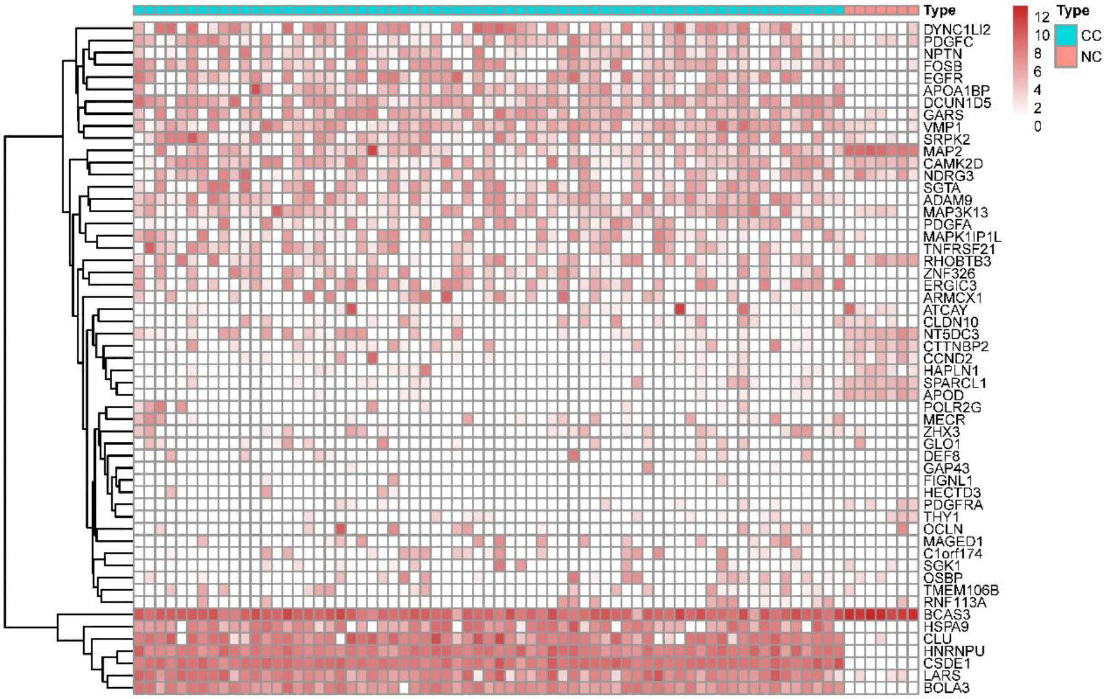

Supplementary Figure S12: Variation in expression of VEGF signaling pathway in cancer and normal single cells population. Heatmaps for each tumor show expression values for VEGF signaling pathway (rows, alphabetically ordered with receptors and ligands paired) in each cell (columns).

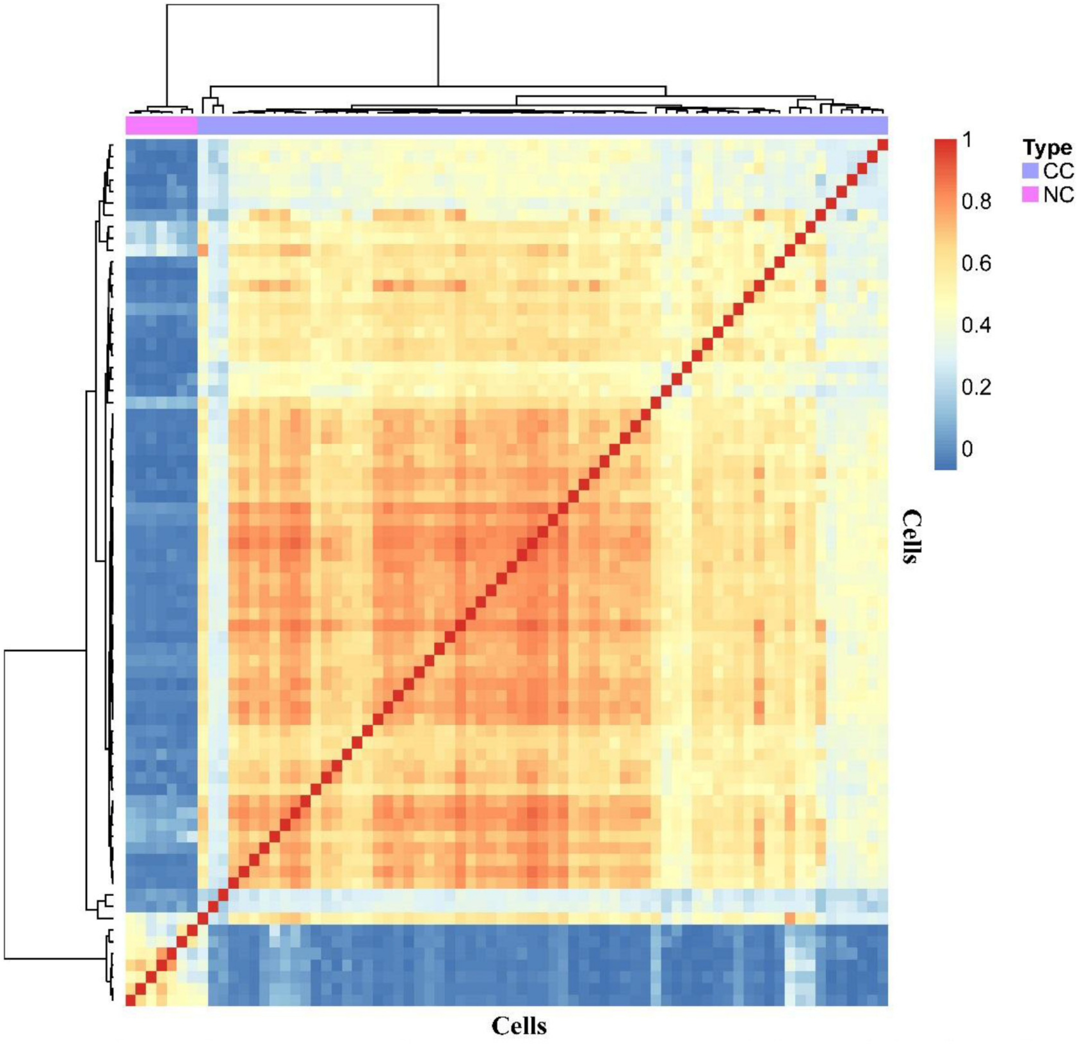

Supplementary Figure S13: Similarity and variation in single cell profiles. Correlation matrix for all 74 cells (67 tumor and 7 normal single cells) was clustered.

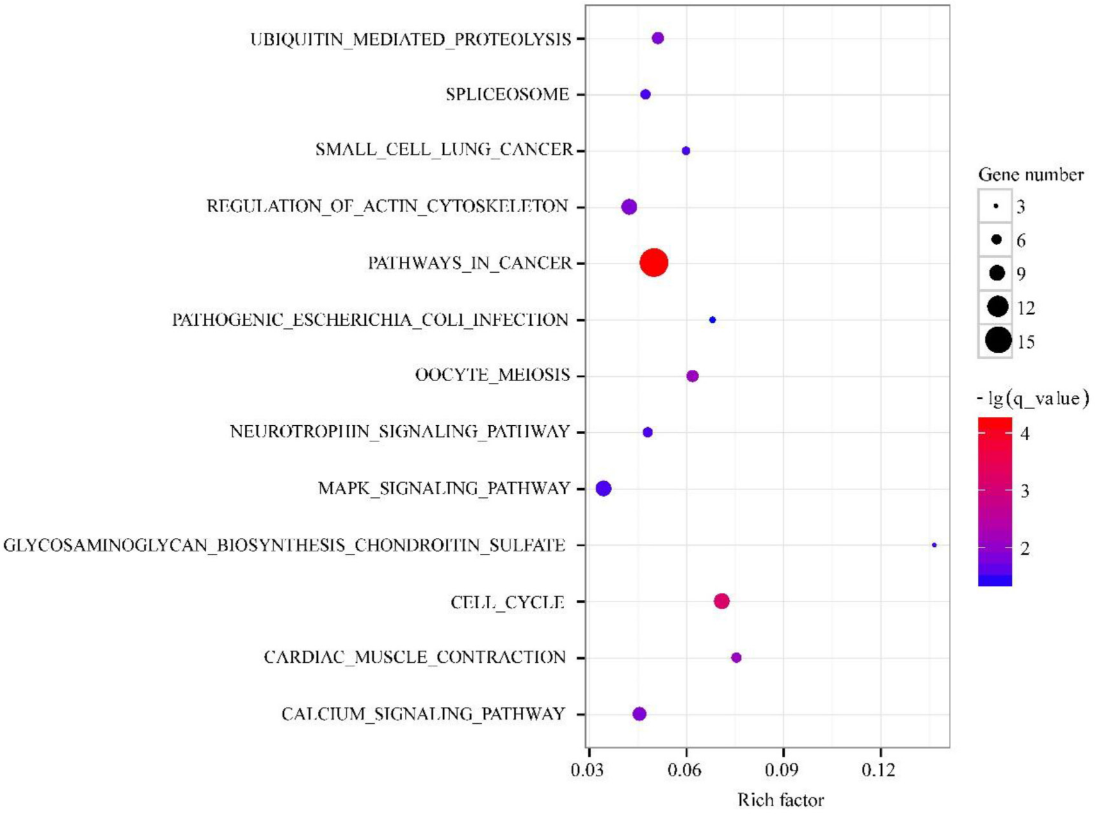

Supplementary Figure S14: Pathways enriched by genes derived from the largest module ("darkorange" module). Size of the dots represents the number of genes and the color represents the q-value.

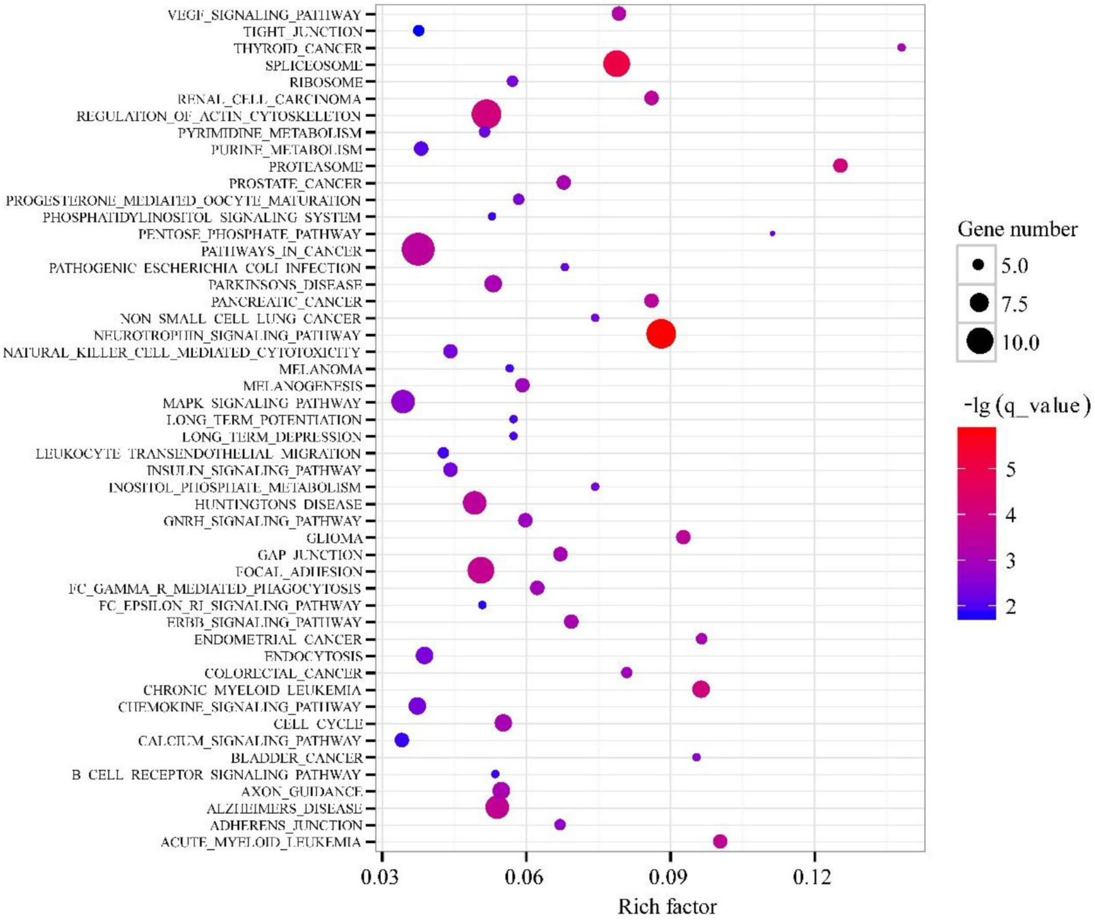

Supplementary Figure S15: Pathways enriched by genes derived from the second largest module ("blue" module). Size of the dots represents the number of genes and the color represents the q-value.

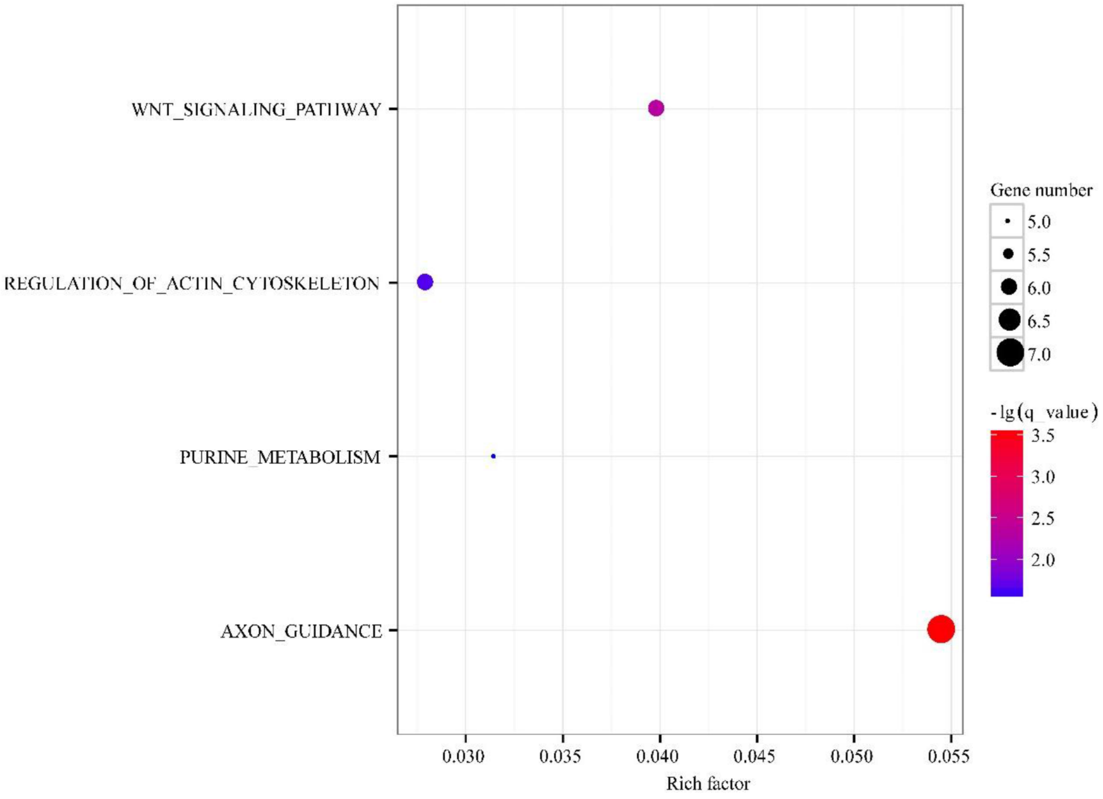

Supplementary Figure S16: Pathways enriched by genes derived from the "yellow" module. Size of the dots represents the number of genes and the color represents the q-value.

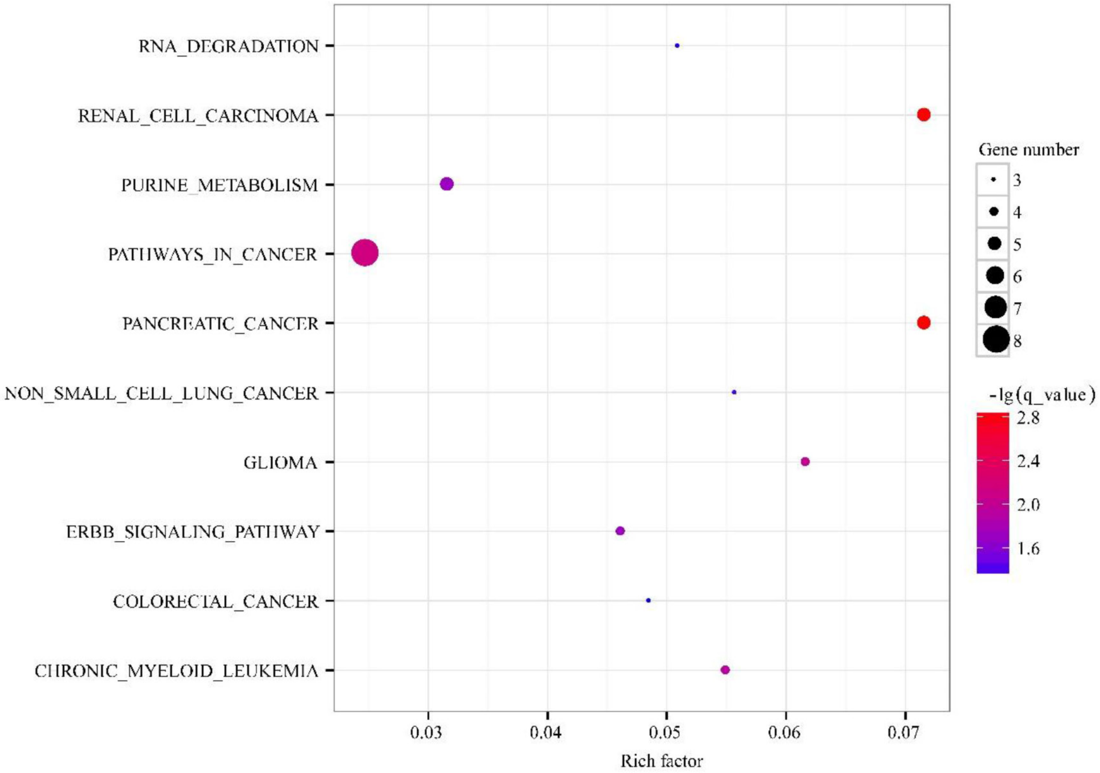

Supplementary Figure S17: Pathways enriched by these genes derived from the "green" module. Size of the dots represents the number of genes and the color represents the q-value.

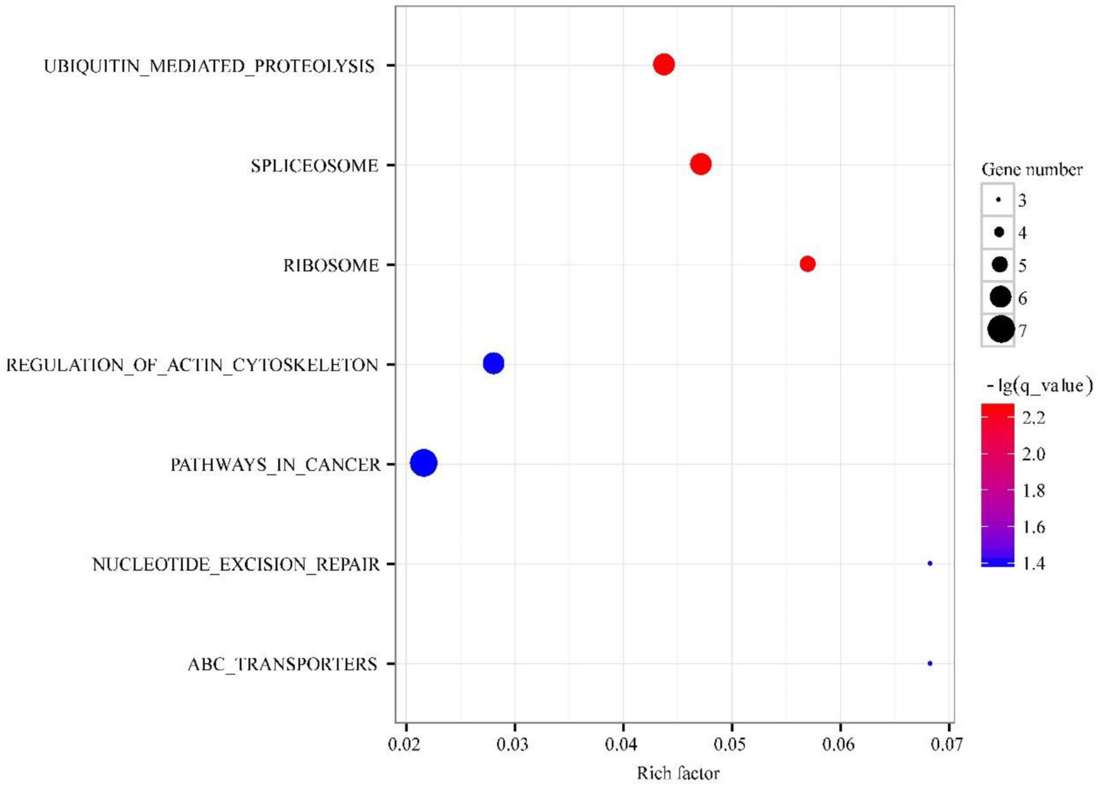

Supplementary Figure S18: Pathways enriched by genes derived from the "brown" module. Size of the dots represents the number of genes and the color represents the q-value.

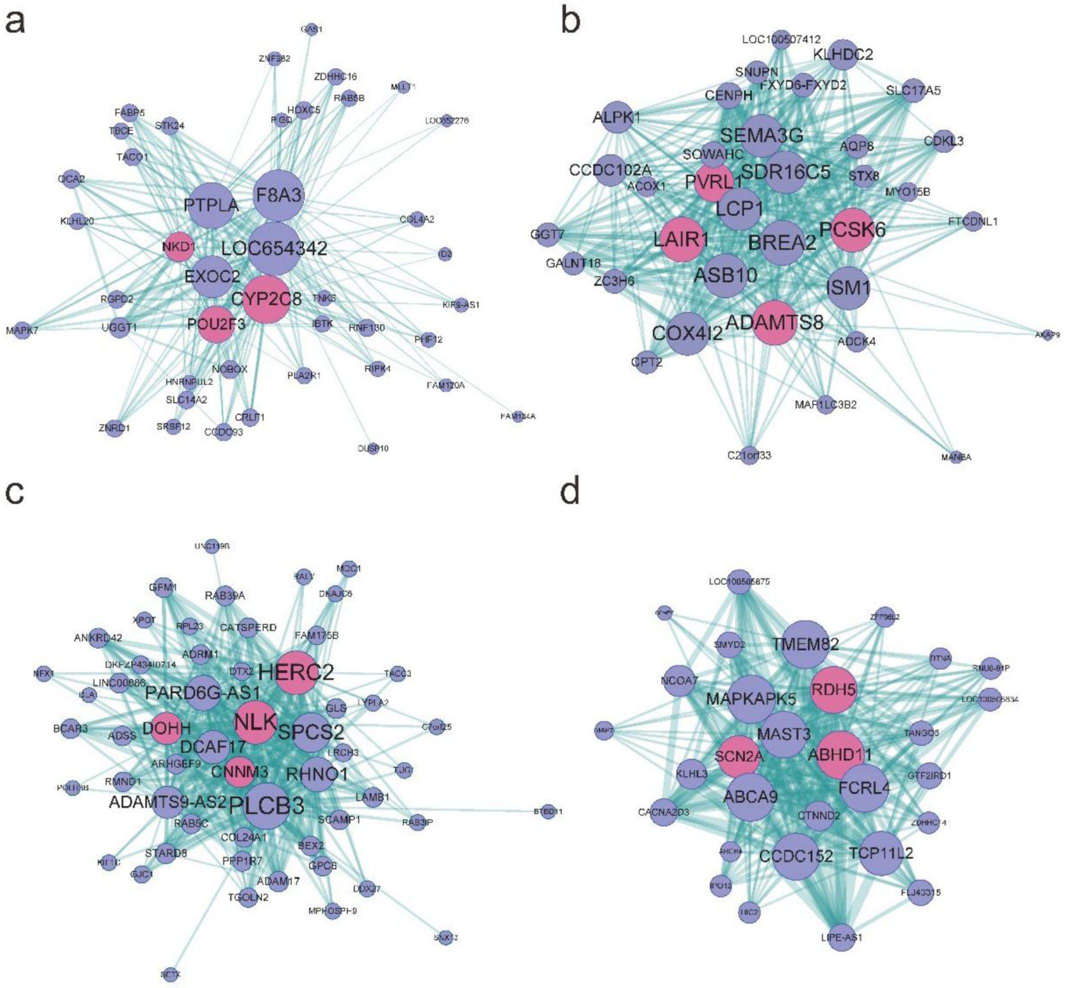

Supplementary Figure S19: Co-expression networks of a. blue **b.** brown **c.** yellow and **d.** green modules. The sizes of the dots represent "hubness" and pink highlights the commonly reported cancer-related genes.

Supplementary Table S1: Details of the data mapping parameters.

See Supplementary File 1

Supplementary Table S2: The overview of these DEGs.

See Supplementary File 2

Supplementary Table S3: The pathways enriched by DEGs.

See Supplementary File 3

Supplementary Table S4: KEGG enrichment of these genes in the top five module individually.

See Supplementary File 4
